# Platelet Depletion Alters MCP‐1 Measurements in Plasma From Children With Dengue

**DOI:** 10.1155/jotm/8409965

**Published:** 2026-05-19

**Authors:** Héctor Felipe Ocampo-Amariles, Sebastián Castro-Trujillo, Carlos F. Narváez

**Affiliations:** ^1^ Medical Program, Faculty of Health Sciences, Universidad Surcolombiana, Neiva, Huila, Colombia, usco.edu.co; ^2^ Department of Pediatrics, Faculty of Health Sciences, Universidad Surcolombiana, Hospital Universitario Hernando Moncaleano Perdomo, Neiva, Huila, Colombia, usco.edu.co

**Keywords:** dengue, MCP-1, plasma-poor platelets, platelets, severe dengue

## Abstract

**Background:**

Platelets are increasingly recognized as contributors to the cytokine storm in dengue, yet their residual presence in plasma may bias cytokine measurements. We compared MCP‐1 levels between paired regular plasma (RP) and platelet‐poor plasma (PPP) in children with dengue and evaluated how depletion influences cytokine quantification.

**Methods:**

We first conducted a systematic review of plasma isolation methods in pediatric dengue studies. We then collected paired RP and PPP from 16 hospitalized children with dengue (11 with warning signs and 5 with severe dengue). The PPP was obtained by double centrifugation and verified to contain < 10,000 platelets/μL. Absolute platelet counts were determined by flow cytometry using an antihuman CD41 antibody and bead‐based quantification. After one freeze–thaw cycle, MCP‐1 and IFN‐γ (negative control for platelet secretion) were measured by cytometric bead array.

**Results:**

Among 167 reviewed articles, only 40% reported plasma isolation methods and 5% performed platelet depletion, with wide variability in centrifugation protocols. In our cohort, 63% of RP samples exceeded the platelet‐poor threshold compared with only 10% of PPP after double‐spin depletion. After effective platelet removal, MCP‐1 levels increased nearly 5‐fold in PPP versus RP (*p* = 0.008), while IFN‐γ showed only a very modest rise, indicating that platelets are highly susceptible to physical and mechanical factors during the depletion process.

**Conclusion:**

Standard plasma isolation fails to consistently remove platelets, and subsequent depletion protocols can artifactually elevate the platelet‐associated cytokines MCP‐1. These findings underscore the need for standardized reporting of platelet depletion methods in dengue immunology research.

## 1. Introduction

Dengue virus (DENV) is a mosquito‐borne *Orthoflavivirus* with four serotypes (DENV‐1 to DENV‐4) that can cause a wide range of clinical manifestations from asymptomatic and mild febrile illness to more severe, life‐threatening forms, especially in children [1]. The incidence of dengue has increased worldwide in recent decades, with up to 390 million infections annually [2], largely attributed to climate change, globalization, and uncontrolled urban growth, among other factors. In Colombia, dengue remains an endemic disease with the simultaneous circulation of all serotypes, with the highest incidence recorded in 2024, with 969.3 cases per 100,000 inhabitants [3].

The pathogenesis of severe dengue (SD) involves a complex interplay of viral, immunologic, and host genetic factors. Among the most studied mechanisms is the cytokine storm—a dysregulated and excessive release of inflammatory mediators that contributes to vascular permeability, plasma leakage, and multiorgan dysfunction [4]. This aberrant immune activation is shaped by innate and adaptive responses and is influenced by prior immunity, serotype, nonstructural viral proteins such as nonstructural protein 1 (NS1), and the timing of infection. Multiple immune cells have been implicated as sources of circulating cytokines, including monocytes and cross‐reactive B and T cells. Notably, platelets have emerged as key immune effector cells in dengue pathogenesis, as activated platelets contribute to inflammation by releasing proinflammatory cytokines such as IL‐1β and MCP‐1 [5–8]. Indeed, plasma levels of cytokines such as IL‐1β, IL‐6, TNF‐α, and MCP‐1 [9, 10] have been consistently associated with disease severity in dengue and may serve as prognostic biomarkers [11–14]. However, a technical limitation in investigating circulating cytokines in dengue has received poor attention: residual platelets in plasma samples may bias cytokine measurements, particularly after freeze–thaw cycles. Residual platelets can artificially elevate cytokine levels such as IL‐1β and soluble CD40 ligand (sCD40L) [6]. Additionally, variations in centrifugation force, processing time, and temperature during plasma isolation significantly influence cytokine quantification [6]. Despite this concern, data on plasma collection and platelet‐depleting protocols are scarce and inconsistently reported across the dengue studies. This is particularly important because cytokine profiles are being explored as immune correlates of vaccine efficacy in DENV and other viral infections. While neutralizing antibody titers remain the primary endpoint in vaccine trials, growing evidence suggests that cytokine signatures could complement the serological assessment of vaccine efficacy [15, 16]. Because sample handling and processing time critically affect platelet activation and survival, inconsistent reporting of these parameters in dengue cytokine studies limits comparability and motivated our evaluation of standardized plasma preparation methods. Thus, assessing the practical impact of plasma purification protocols and residual platelets within the pathophysiological context of DENV infection, where cytokine quantification is most clinically relevant, is an important area for exploration.

To address this gap, we initially conducted a systematic review to evaluate plasma isolation methods and the prevalence of platelet depletion in studies evaluating circulating cytokines in pediatric dengue. Then, we assessed the influence of residual platelets on plasma cytokine levels. To do this, we adapted a widely used hematological method to deplete platelets and compare the circulating levels of platelet‐related cytokine MCP‐1 between paired regular plasma (RP) and platelet‐poor plasma (PPP) in hospitalized children with confirmed DENV infection.

## 2. Methods

### 2.1. Ethical Considerations

This study was approved by the Ethics Committee of the Hospital Universitario de Neiva (approval code no. 02‐07 2023). Written informed consent was obtained from parents, and informed assent was obtained from children aged 7 years or older prior to participation. All procedures adhered to the principles outlined in the Declaration of Helsinki. The study included hospitalized pediatric patients with confirmed dengue. Sample collection occurred at the Hospital Universitario de Neiva in Southern Colombia between June and July 2024, during the largest dengue epidemic ever recorded in the Americas.

### 2.2. Systematic Review

**FIGURE 1:**
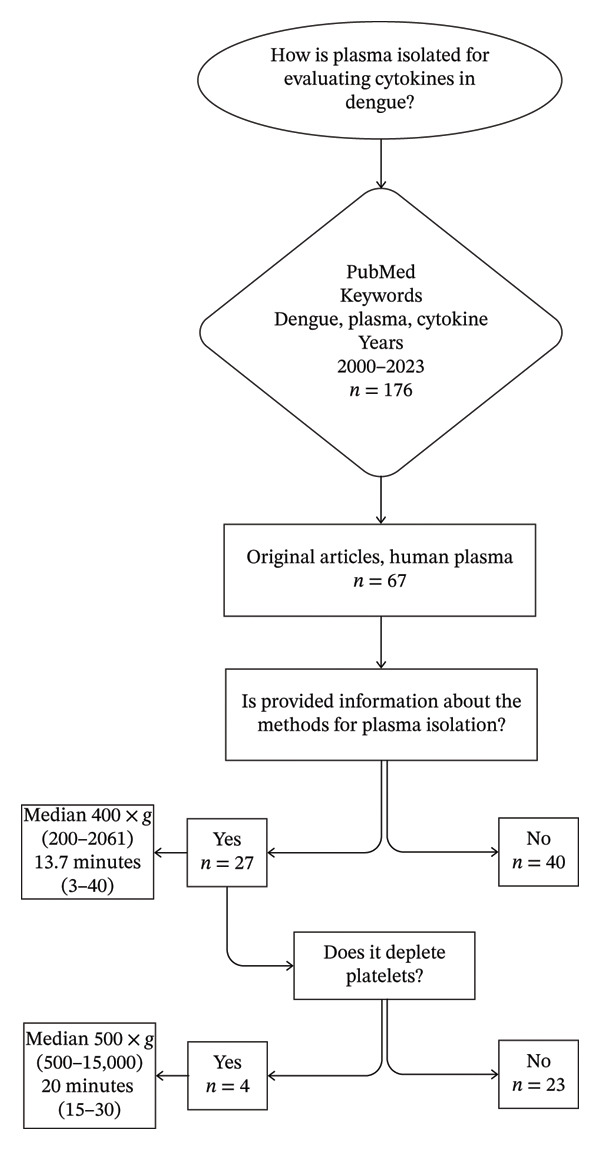
Systematic review flowchart summarizing the selection process of studies evaluating plasma isolation methods in dengue research. The search strategy was conducted in PubMed using the keywords “dengue,” “plasma,” and “cytokine” to identify 176 records published between 2000 and 2023. After screening, 67 original articles focusing on human plasma were selected. Among these, 27 studies provided details on plasma isolation methods, while 40 did not. The diagram also displays the reported centrifugation parameters, showing median values with ranges for relative centrifugal force (× *g*) and centrifugation time (minutes). Only four studies described platelet‐depleting protocols.

Initially, to analyze different protocols for isolating plasma and reducing residual platelets in plasma from children with dengue, a systematic review was conducted in accordance with the Preferred Reporting Items for Systematic Reviews and Meta‐Analyses (PRISMA) guidelines [17]. MeSH terms and keywords related to “dengue,” “platelets,” and “cytokines” were combined for the search, which was performed in PubMed, encompassing English articles published between 2000 and the end of 2023. This specific period was selected because cytokine measurements became widely used in dengue research after 2000, and this period spans both the 1997 and 2009 World Health Organization (WHO) classifications, allowing us to assess reporting trends over time. The inclusion criteria focused on original research articles that evaluated DENV‐infected patients confirmed by laboratory tests, such as NS1 or DENV‐IgM in plasma. The information targeted from each study included at least the plasma preparation method (× *g* and timing) and if platelet depletion was performed. Additional information included country, sample size, age of the individuals, anticoagulant type, and cytokines measured. Reviews, meta‐analyses, animal model studies, in vitro studies, and studies lacking evaluation of plasma cytokines were excluded. The article selection process consisted of two phases. First, titles and abstracts were reviewed to identify relevant publications. Then, a full‐text assessment was conducted to confirm their eligibility. Figure 1 shows the flowchart for the systematic review.

### 2.3. Patients and Samples

This study prospectively included 16 symptomatic children (5 months–12 years of age) diagnosed with DENV infection who attended the pediatrics department at the Hospital Universitario de Neiva (a reference center for dengue with warning signs [DWS] and SD) in Southern Colombia, during June–July 2024. Children with suspected dengue based on clinical criteria (fever plus two or more of the following: headache, retro‐orbital pain, myalgia, arthralgia, rash, warning signs, hemorrhagic manifestations, or vascular leakage) were identified by the attending pediatrician. Peripheral venous blood samples were collected on admission in tubes containing ethylenediaminetetraacetic acid (BD Vacutainer EDTA, cat no. 367863, San Jose, California) and assessed within 4 h after bleeding.

For the diagnosis, classification, and clinical monitoring of children with dengue, the revised 2009 WHO guidelines were followed [18]. These guidelines categorize DENV‐infected patients into dengue without warning signs (DNS), DWS, and SD. This study included 11 children with DWS and 5 with SD. DENV infection was confirmed by detecting viral NS1 and/or DENV‐specific immunoglobulin M (IgM) in plasma using commercially available ELISA kits (Dengue early NS1 kit, cat no. 01PE40, and capture IgM dengue ELISA kit, cat no. 01PE20, both from Abbott), following the manufacturer’s instructions. Children with DNS (mild dengue) were not included because the Hospital Universitario de Neiva, where the study was conducted, is a high‐complexity referral center that mainly manages DWS and SD cases. Consequently, most mild cases are treated at outpatient or lower‐level facilities and are not eligible for hospital‐based enrollment. Detailed clinical and laboratory results for the enrolled children are available in the Supporting Information (available here).

### 2.4. Isolation of Plasma and Application of the Method for Platelet Depletion

To obtain RP, we used the median centrifugation parameters reported in the dengue literature, as selected for the systematic review (400 × *g* for 10 min; Figure 1). For paired PPP, we followed ICSH coagulation guidelines, using a widely used hematological protocol [19]. Plasma obtained from whole blood after centrifugation at 1200 × *g* for 10 min was classified as RP according to internationally standardized protocols [19]. Additionally, a two‐step isolation method was employed to obtain a paired PPP. The whole blood was centrifuged at 2000 × *g* for 10 min, followed by a second centrifugation of the resulting plasma under the same conditions. As previously established, the PPP was defined as having a platelet count below 10,000 cells/μL [20]. The total processing time was 20 min. We performed all centrifugations at room temperature (26°C).

The depletion of platelets in plasma was confirmed through absolute platelet counting using a bead‐based assay on cells expressing CD41, a surface marker of mature human platelets, as described below. We estimated leukocyte contamination by analyzing side scatter and the CD41 cell surface marker expression (excluding beads) by flow cytometry. In this analysis of PPP, a median 28.6% range (0.74–83.5) of events were CD41^+^, corresponding to the platelet population, suggesting that in the PPP, approximately a median (range) 61% (10.2–76.7) of the CD41^-^ events may represent leukocytes or other cellular debris such as microvesicles. After platelet counting, all plasma samples were stored at −80°C for two weeks and subsequently thawed for cytokine analysis, ensuring that both RP and PPP underwent an identical single freeze–thaw cycle.

### 2.5. Absolute Platelet Quantification Using Bead‐Based Flow Cytometry

We measured platelet concentration in plasma using a bead‐based absolute counting method with BD Trucount beads (BD Biosciences, cat no. 340334, San Diego, CA). This approach relies on fluorescent beads of known concentration, serving as an internal reference to determine the absolute number of platelets in each sample volume. Platelet quantification was performed using anti‐human CD41 antibodies conjugated to phycoerythrin (BioLegend, cat no. 303706). In the analysis, a fixed volume of fluorescent beads was added to each plasma sample, enabling the identification of platelet events via forward scatter, side scatter, and CD41 expression. The ratio of platelet‐to‐bead events was used to calculate absolute platelet counts in RP and PPP. This method ensures accurate, reproducible quantification while confirming the effectiveness of the platelet‐depleting technique.

The results were analyzed using a FACSCanto II cytometer and the FACS DIVA (v.3.0, BD Biosystem) flow cytometry software. A gating strategy was applied to distinguish platelets from debris and other cellular fragments. The platelet counts obtained in RP and PPP were compared to confirm the efficiency of the depletion protocol.

### 2.6. Cytokine Quantification

After one thaw cycle of RP and PPP, the levels of MCP‐1 and IFN‐γ cytokines were quantified using Cytometric Bead Array (CBA) assays: Human MCP‐1 Flex Set and Human IFN‐γ Enhanced Sensitivity Flex Set (BD Biosciences, cat No. 558287; cat No. 561515, respectively, San Diego, CA). The detection limits for MCP‐1 and IFN‐γ (in pg/mL) were 0.3 and 0.15. All manufacturer recommendations were followed for cytokine detection. MCP‐1 is a platelet‐related chemokine implicated in vascular leakage and disease severity during dengue, making it an ideal analyte to assess the impact of residual platelets on cytokine measurement. On the other hand, IFN‐γ, secreted mainly by T and NK cells, served as a negative control for platelet secretion, as platelets do not secrete this cytokine.

### 2.7. Statistical Analysis

The statistical analysis was performed using GraphPad Prism Software v. 9 (GraphPad Software, San Diego, CA, USA). Data are presented as medians and ranges. The Mann–Whitney *U* test was used to compare continuous variables between two independent groups, while Fisher’s exact test was applied to categorical variables. The Wilcoxon exact test was used to compare cytokine levels between paired RP and PPP from the same children. Correlations between cytokine levels and other variables were assessed using the Pearson coefficient (r) and Spearman test (rho). In all cases, a *p* value < 0.05 was considered statistically significant. For statistical purposes, cytokine concentrations below the assay detection threshold were imputed as one‐half of the limit of detection.

## 3. Results

### 3.1. Incomplete Report and Methodological Variability in Plasma Obtention Protocols Among Dengue Studies

Variability of plasma isolation methods and platelet depletion may influence the assessment of plasma cytokines. We identified 176 references and selected 67 original articles analyzing cytokines in human plasma from 1991 patients with laboratory‐confirmed dengue. Of these, 34% (23/67) and 6% (4/67) of studies described plasma isolation and a detailed platelet‐depleting protocol, respectively. 60% (40/67) did not specify the methods used. The reported median centrifugation force was 400 ×  , ranging from 200 to 2061 × *g*, and the times ranged from 3 to 40 min. Overall, the systematic review identified methodological inconsistencies in plasma preparation across dengue cytokine studies, which directly informed the design of our prospective analysis to experimentally evaluate the impact of platelet removal on cytokine quantification. Figure 1 shows the flowchart of the systematic review.

### 3.2. Epidemiological, Clinical, and Laboratory Characteristics of the Study Population

Children with SD exhibited distinct clinical and laboratory profiles compared to those with DWS. Hepatomegaly and elevated liver enzymes are common in SD, while platelet counts decline significantly in that group. These findings are consistent with the clinical classification of dengue, supporting its association with disease severity [4, 21]. Table 1 summarizes the epidemiological, clinical, and laboratory characteristics of children diagnosed with dengue, categorized into those with DWS and those with SD.

**TABLE 1:** Clinical and laboratory characteristics of children diagnosed with dengue.

Epidemiological data	DWS (*n*) = 11	SD (*n*) = 5	*p* value
Epidemiological data			
Age (years), median (range)	7 (4–11)	8 (0.41–12)	0.846
Men/women	7/4	2/3	0.596
Days of symptom onset, median (range)	5 (3–6)	6 (5–6)	**0.0007**
Clinical manifestations (*n*)			
Fever	11	5	0.999
Rash	4	4	0.282
Abdominal pain	8	5	0.999
Hepatomegaly	4	5	**0.033**
Vascular leakage (pleural effusion > 25%)	0	3	**0.017**
Organ involvement (liver damage)	0	2	0.083
Laboratory tests, median (range)			
Serum albumin (g/dL)	3.8 (2.83–4.59)	4.1 (2.5–12.6)	0.059
Partial thromboplastin time (Seg)	34 (31–38.5)	47 (23.4–69)	0.539
Aspartate aminotransferase (IU/L)	128.3 (37.4–245)	390 (106–1415)	**0.001**
Alanine aminotransferase (IU/L)	65.5 (36.5–260)	256 (98.2–754)	**0.038**
C‐reactive protein (mg/dL)	0.5 (0.31–6.51)	1.2 (0.8–2.8)	0.385
Number of cells/mm^3^, median (range)			
Leukocytes	8230 (3450–9660)	6990 (5150–8380)	0.763
Platelets	89,000 (26,000–129,000)	20,000 (11,000–42,000)	**0.030**

*Note:* Categorical and continuous variables were analyzed using Fisher’s exact and Mann‐Whitney U tests, respectively.

Abbreviations: DWS, dengue with warning signs; SD, severe dengue.

### 3.3. PPP Was Obtained Through a Double‐Centrifugation Protocol

When we stained RP samples with anti‐human CD41‐PE, a platelet‐specific marker, a median of 74.8% range (2.5–94.3) of the events corresponded to platelets, confirming the predominance of these cells in conventional plasma. Of note, we used threefold higher centrifugation forces than those studies evaluated through the systematic review (1200 × *g* compared to 400 × *g*), indicating a possible underreporting of remaining platelets in those studies. After applying the ICSH‐recommended double‐centrifugation protocol, the CD41^+^ population decreased to a 5.3% range (0.8–36.6), suggesting a substantial reduction in platelet content (Figure 2(a)). We analyzed the platelet‐to‐leukocyte ratio (P:L) in both conditions. The medians (range) of the P:L for PPP and RP were 3 (0.36–83) and 24 (11–36), respectively (*p* = 0.002, Wilcoxon test). We quantified platelet concentration using a bead‐based absolute counting assay to validate these findings. The median (range) of the CD41+ cells was 22,779 (2541–94,398) and 1009 (134–12,147) in RP and PPP, respectively (*p* < 0.001, Wilcoxon test), demonstrating a depletion of the 96% of the CD41+ events after the double‐centrifugation protocol (Figure 2(c)). Furthermore, only 36% of the RP met the PPP criterion (< 10,000 platelets/μL). In contrast, 91% of the PPP assessed by double centrifugation reached this threshold, demonstrating the method’s effectiveness in reducing platelet contamination (Figure 2(c)). Finally, we confirmed a strong positive correlation between platelet count and CD41 expression (*r* ^2^ = 0.88, *ρ* = 0.95, *p* < 0.001), supporting CD41 as a consistent marker of human platelet content (Figure 2(b)). These results support the double‐centrifugation protocol as a reproducible method for depleting platelets in plasma from pediatric patients with dengue.

FIGURE 2Platelet depletion in children with dengue. Flow cytometry was used to evaluate platelet depletion efficiency in regular plasma (RP) compared with platelet‐poor plasma (PPP). The analysis employed BD Trucount beads as an internal reference to determine absolute platelet counts. (a) The first panel (upper left) represents RP, where beads appear above, as a distinct population. The second panel (upper right) shows RP stained with the platelet‐specific marker CD41, identifying mature platelet events. The third panel (lower left) shows the forward and side scatter of PPP, where scatter plots indicate a lower density of platelet events than RP. The fourth panel (lower right) corresponds to PPP and shows a reduced platelet population following the two‐step centrifugation protocol. (b) A correlation analysis between platelet counts and CD41^+^ percentage revealed a strong positive association, confirming the relationship between platelet abundance and the CD41^+^ signal. (c) A comparative scatter plot shows absolute platelet counts in paired PR and PPP, with a significant reduction in platelet concentration in PPP. The discrete distribution reflects integer bead‐based counting (Trucount method), and 12 pairs are represented (some *x*‐axis values overlap visually). *p* value of the Pearson, Spearman, and Wilcoxon tests is shown.

**Figure:**
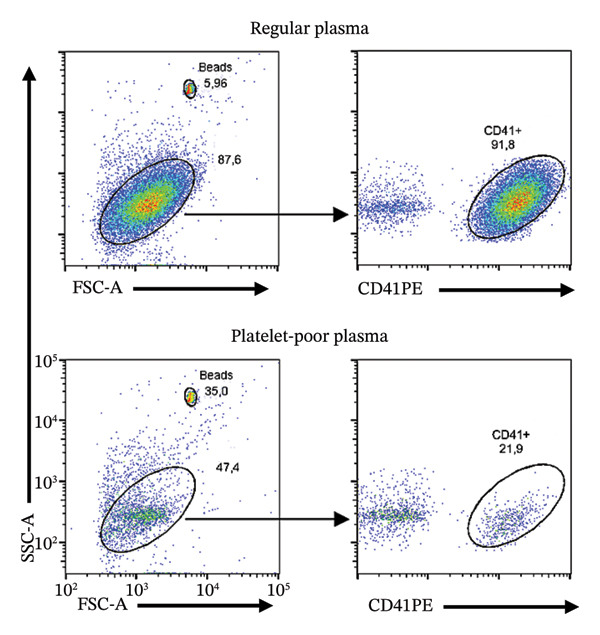
(a)

**Figure:**
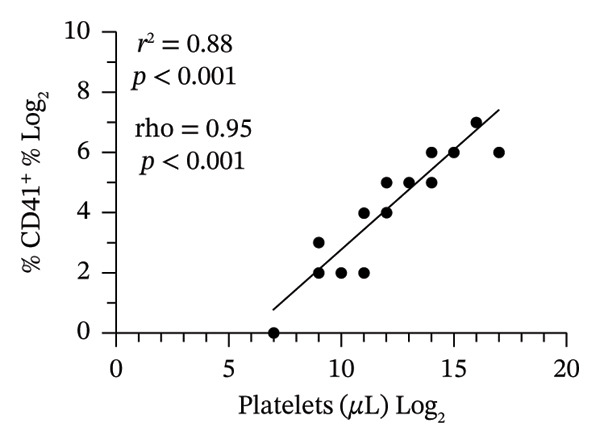
(b)

**Figure:**
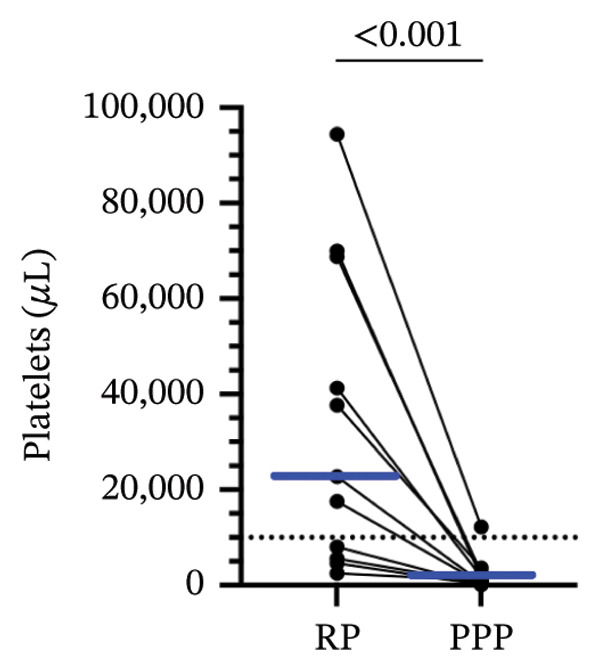
(c)

### 3.4. Higher Proinflammatory Cytokine Levels in PPP Despite Lower Platelet Counts

Next, we compared the median concentrations of plasma MCP‐1 (secreted by platelets) and IFN‐γ (secreted by T and NK cells) between RP and PPP. The median (range) of MCP‐1 in RP and PPP was 7 pg/mL (1.5–182.8) and 36 pg/mL (1.5–375.6) (*p* = 0.008, Wilcoxon test), while that for IFN‐γ was 0.1 pg/mL (0.1–17.6) and 0.2 pg/mL (0.1–23.1) (*p* = 0.04, Wilcoxon test). Thus, MCP‐1 increased nearly 5‐fold in PPP compared to RP (Figure 3(a)). Conversely, there was a slight increase of 1‐fold in IFN‐γ (Figure 3(b)). These findings may be related to the lower P:L in PPP compared to RP, as it is known that the platelet and leukocyte rates can enhance mutual cellular activation, although other factors may also be involved. Also, we performed correlation analysis and found that platelet counts and MCP‐1 levels in PPP and RP do not correlate (Spearman’s rho = 0.64, *p* = 0.06; rho = 0.16, *p* = 0.67, respectively).

FIGURE 3Levels of monocyte chemoattractant Protein 1 (MCP‐1) (a) and interferon‐gamma (IFN‐γ) (b) in regular plasma (RP) and platelet‐poor plasma (PPP). Whole blood obtained from children with dengue (*n* = 16) was single (to RP) or double‐centrifuged (to obtain PPP). Paired RP and PPP were frozen for 2 weeks, and then a bead‐based flow cytometry assay was performed to evaluate their concentrations. Dashed lines bound paired RP and PPP samples. Solid lines represent the median. The dotted line represents the detection limit of the assay. ^∗^ *p* = 0.04 and ^∗∗^ *p* = 0.008, Wilcoxon test for paired samples.

**Figure:**
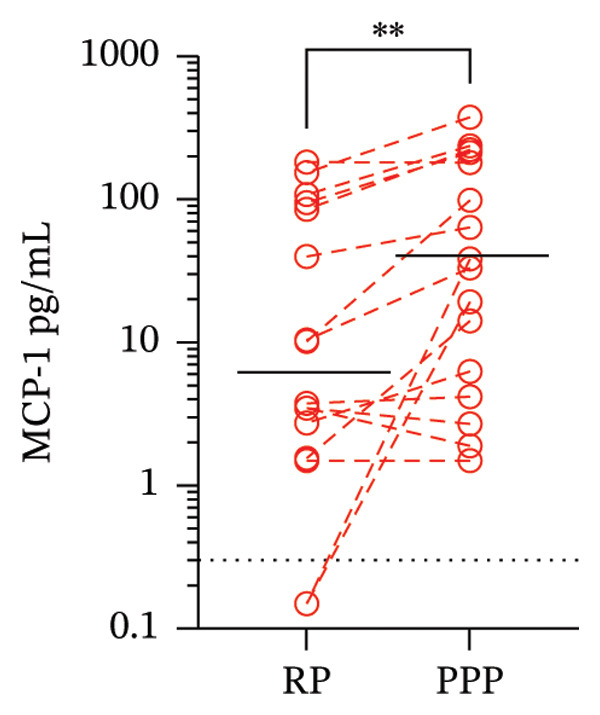
(a)

**Figure:**
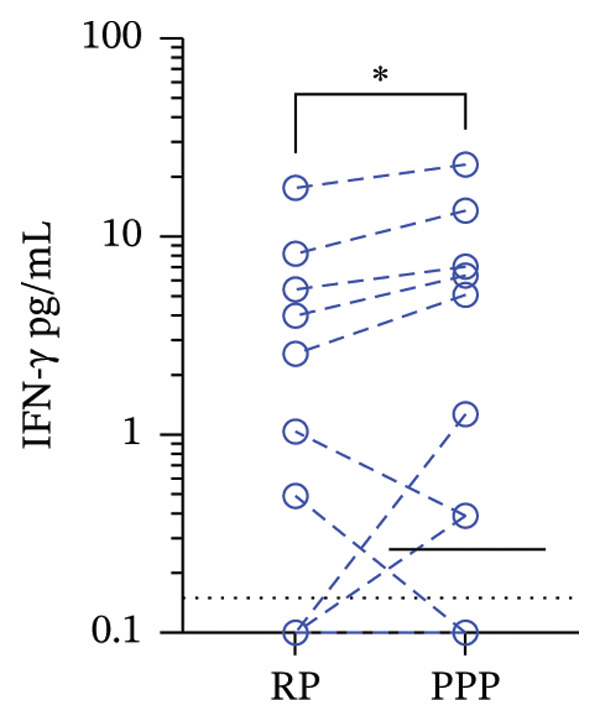
(b)

## 4. Discussion

Evaluation of circulating cytokines in dengue is key for identifying prognostic markers, therapeutic targets, and potential correlates of vaccine efficacy. Our findings highlight potential sources of bias in plasma cytokine assessment, including residual platelets and variable depletion protocols. Throughout the systematic review, we found that most articles assume default plasma separation without providing detailed protocols, increasing procedural variability, and a few studies deplete platelets. Additionally, our results show a high residual platelet count after standard plasma isolation. The method used to deplete platelets was optimal, but it significantly affects the evaluation of circulating cytokines in children with dengue.

The number of platelets could influence cytokine levels, as previous studies have shown elevated levels of growth factors, proinflammatory, and anti‐inflammatory cytokines in platelet‐rich plasma [22, 23]. However, in our study, MCP‐1 levels increased after the platelet depletion. Evidence suggests that hypergravity can induce cellular activation [24, 25], as higher centrifugation speed and duration activate platelets, leading to increased expression of P‐selectin, a key marker of platelet activation [26]. This could explain the increase in MCP‐1 plasma levels after platelet depletion, as an additional step applied to the PPP could favor the release of granular contents, including growth factors and cytokines [8]. The absence of a clear correlation between baseline platelet counts and MCP‐1 changes suggests that additional factors beyond platelet number, including processing‐related activation or cell damage, likely contribute to MCP‐1 release during depletion. Still, the slight increase in IFN‐γ was unexpected, as it originates from NK cells, as well as CD4^+^ and CD8^+^ T cells [27], suggesting some minimal degree of leukocyte contamination in plasma. Additionally, the elevated cytokine levels in PPP could stem from changes in the leukocyte‐to‐platelet ratio following platelet depletion. Platelets can modulate immune responses through direct and indirect mechanisms. The exposure of platelet P‐selectin on the surface binds to PSGL‐1 receptors on monocytes and neutrophils, inducing the release of cytokines including IL‐1β, IL‐8, and TNF‐α [28]. On the other hand, activated platelets during DENV infection release soluble factors, such as PF4, MCP‐1, RANTES, and CXCL8, as well as extracellular vesicles, which in turn recruit leukocytes to the site of inflammation and stimulate their activation [8, 29, 30]. This suggests that activated or damaged platelets may stimulate cytokine production in PPP through various leukocyte activation pathways, which is likely intensified by changes in the leukocyte‐to‐platelet ratio induced by the isolation methods. Also, increasing evidence indicates that DENV can infect bone marrow megakaryocytes, leading to altered platelet production and activation, which in turn may contribute to the release of chemokines and cytokines from both megakaryocytes and derived platelets, further complicating the interpretation of plasma cytokine profiles during acute infection [31].

The International Council for Standardization in Hematology recommends isolating plasma at 1700 g for 10 min to obtain PPP [20], followed by a double spin to ensure the samples are sufficiently platelet‐poor. Yet, we observed poor adherence to this recommendation, with high variability and limited description of plasma isolation methods in dengue studies, which influence the reproducibility and external confirmation of findings. Despite the low circulating platelet count during dengue, especially in severe cases, we observed a significant number of remaining platelets above the recommended threshold (10,000 cells/μL), and their depletion by double‐spinning increased MCP‐1 levels. Thus, given that these guidelines are designed for hemostatic tests, more studies are needed to evaluate alternative platelet‐depleting methods in immunology studies. In addition, our findings underscore the potential relevance of residual platelets in other clinical contexts, such as transfusion medicine, where activated platelets in fresh frozen plasma may contribute to febrile reactions or acute lung injury [32].

This study has limitations, including a small sample size, the absence of healthy control and adult samples, and the lack of measurement of specific platelet activation markers, such as soluble P‐selectin and CD40L [33, 34], which reduced the ability to confirm platelet activation. Additionally, we measured only one nonplatelet cytokine (IFN‐γ) as a negative control. Our findings underscore the potential effect of remaining platelets in other types of studies and clinical contexts, as remaining platelets in plasma can bias the measurement of extracellular microRNAs or induce transfusion‐related fever or acute lung injury in patients receiving fresh frozen plasma [35, 36]. Future work comparing multiple preparation protocols will be essential to assess cytokine stability and platelet‐derived contributions under real‐world conditions. Thus, this work should be interpreted as a proof‐of‐concept study designed to experimentally demonstrate how plasma preparation and residual platelets could influence cytokine measurements in pediatric dengue, rather than to provide definitive clinical cutoffs or broadly generalizable estimates. We therefore highlight the need for larger, multicenter studies that include diverse age groups, clinical severities, and standardized preanalytical protocols.

This study demonstrates that residual human platelets do not significantly bias circulating MCP‐1 measurements in pediatric dengue. We recommend the development and standardization of new depletion methods because current protocols across the literature are highly variable and inconsistently reported, which can introduce methodological bias, limit reproducibility, and may affect results in other populations or for less platelet‐specific cytokines.

## 5. Conclusion

Platelet depletion can elevate MCP‐1 levels in plasma from children with dengue. The wide variability in current methods and the potential for such artifacts underscore the need for standardized protocols and reporting to ensure reproducibility in dengue immunological studies.

## Author Contributions

Héctor Felipe Ocampo‐Amariles and Sebastián Castro‐Trujillo enrolled patients. Héctor Felipe Ocampo‐Amariles and Sebastián Castro‐Trujillo performed the experiments, the primary data analysis, and the figures. Héctor Felipe Ocampo‐Amariles, Sebastián Castro‐Trujillo, and Carlos F. Narváez wrote the manuscript. Carlos F. Narváez was responsible for funding acquisition.

## Funding

This project was supported by the Vicerrectoría de Investigación, Universidad Surcolombiana, grant no. 3660 (to Carlos F. Narváez).

## Disclosure

All authors approved the final version of the manuscript.

## Conflicts of Interest

The authors declare no conflicts of interest.

## Supporting Information

Additional supporting information can be found online in the Supporting Information section.

## Supporting information


**Supporting Information** A Supporting Excel file containing clinical and laboratory information for the 16 children with confirmed dengue who were enrolled, including days after fever onset, plasma liver enzyme (AST and ALT) values, and complete blood cell counts, is presented. Also, the absolute values (pg/ml) of MCP‐1 and IFN‐*γ* obtained in regular plasma (RP) and platelet‐poor plasma (PPP) for each patient were included.

## Data Availability

The data that support the findings of this study are available from the corresponding author upon reasonable request.
